# Preoperative Imaging Evaluation after Downstaging of Pancreatic Ductal Adenocarcinoma: A Multi-Center Study

**DOI:** 10.3390/cancers11020267

**Published:** 2019-02-25

**Authors:** Alessandro Beleù, Angela Calabrese, Giulio Rizzo, Paola Capelli, Nicolò Bellini, Simona Caloggero, Roberto Calbi, Paolo Tinazzi Martini, Riccardo De Robertis, Giovanni Carbognin, Giovanni Marchegiani, Aldo Scarpa, Roberto Salvia, Claudio Bassi, Mirko D’Onofrio

**Affiliations:** 1 Department of Radiology, G.B. Rossi Hospital, University of Verona, 37134 Verona, Italy; ale.beleu@gmail.com (A.B.); giulioriz11@gmail.com (G.R.); bellini.nico@live.it (N.B.); 2 Department of Radiology, Istituto Oncologico Giovanni Paolo II, 70124 Bari, Italy; acalabrese22@gmail.com; 3 Department of Pathology, G.B. Rossi Hospital, University of Verona, 37134 Verona, Italy; paolacapelli@hotmail.com (P.C.); aldo.scarpa@univr.it (A.S.); 4 Department of Radiology, G. Martino Hospital, University of Messina, 98125 Messina, Italy; simona.caloggero@hotmail.it; 5 Department of Radiology, Ospedale Generale Regionale “F. Miulli”, 70021 Acquaviva della Fonti, Italy; calbi.roberto@gmail.com; 6 Department of Radiology, Ospedale P. Pederzoli, 37019 Peschiera del Garda, Italy; paolo.tinazzimartini@univr.it; 7 Department of Radiology, Ospedale Civile Maggiore Borgo Trento, Azienda Ospedaliera Universitaria Integrata, 37134 Verona, Italy; riccardo.derobertislombardi@univr.it; 8 Department of Radiology, Ospedale “Sacro Cuore, Don Calabria”, 37024 Negrar, Italy; giovanni.carbogni@univr.it; 9 Department of Surgery, G.B. Rossi Hospital, University of Verona, 37134 Verona, Italy; giovanni.marchegiani@aovr.veneto.it (G.M.); roberto.salvia@univr.it (R.S.); claudio.bassi@univr.it (C.B.)

**Keywords:** pancreas, pancreatic cancer, adenocarcinoma, downstaging, CT, chemotherapy

## Abstract

Introduction: Evaluation of pancreatic ductal adenocarcinoma (PDAC) after chemoradiotherapy downstaging is challenging due to computed tomography (CT) overestimation of tumor extension and residual vascular involvement, limiting access to surgery to some patients with potentially resectable tumors. With this study, we wanted to assess which radiological findings are most reliable at pre-operative imaging in the evaluation of PDAC after chemoradiotherapy in order to achieve complete resection. Methods: We retrospectively enrolled 71 patients with locally advanced and borderline resectable PDAC who underwent neoadjuvant chemoradiotherapy. Pre-operative CT or magnetic resonance (MR) have been evaluated by three radiologists to assess major qualitative and quantitative parameters of lesions. Accuracy, sensitivity, and specificity compared to anatomopathological results were evaluated for each parameter. Cohen’s K-coefficient has been calculated to evaluate the inter-observer agreement (IOA). Both single and consensus lecture have been tested. Different dimensional cut-offs were tested to categorize tumors according to their major axis and to compare with anatomopathological diameter, tumor persistence, and margin infiltration. Results: A 25 mm cut-off was 67% sensitive, 90% specific, and 77% accurate in assessing real tumor dimension. 25 mm cut-off reported a 64% sensitivity, 78% specificity, and 69% accuracy in assessing R0 resection. Each 5 mm increment of major axis dimension there is an odds ratio (OR) 1.79 (95% CI 1.13–2.80, *p* = 0.012) for R+ resection. Imaging presence of the perivascular cuff is not associated with tumor persistence and resection margin infiltration (*p* = 0.362). Lesion enhancement and pattern homogeneity were not accurate in determining tumor persistence. IOA was generally poor to fair, except for >25 mm cut-off classification where IOA was moderate. Diagnostic accuracy is superior in consensus lecture rather than single lecture. Conclusion: Imaging methods tend to underestimate PDAC resectability after neoadjuvant-CRT. IOA is poor to fair in evaluating most of the qualitative parameters of downstaged pancreatic adenocarcinoma. Surgery should be considered for downstaged borderline resectable PDACs, independently from perivascular cuff presence, especially for tumors smaller than 25 mm.

## 1. Introduction

Pancreatic ductal adenocarcinoma (PDAC) represents one of the most common causes of cancer-related death in the United States and Europe, with a life expectancy of about 5% at 5 years [1,2]. In fact, PDAC usually presents in an advanced stage of disease at diagnosis with diffuse involvement of major vessels and growth beyond pancreatic borders or metastases, which makes only 20% of cases primarily resectable [3]. Among unresectable PDAC, 70% presents already in stage IV at diagnosis and then can only benefit from palliation [4]; however, 30% of unresectable PDAC includes locally advanced and borderline resectable cancers, which after successful neoadjuvant chemoradiotherapy (CRT) downstaging can be suitable for radical resection (R0), which remains the only curative therapeutic option in order to avoid local recurrence [5,6]. Morbidity and survival of R0 resection after neoadjuvant CRT is similar to patients R0 resectable at diagnosis [7], with approximately one-third of initially non-resectable tumors became resectable after neoadjuvant CRT [8,9,10]. Neoadjuvant CRT is then indicated in doubtful cases or when the R1 resection risk is high [2]. The main advantages of neoadjuvant CRT are to achieve local tumor reduction, early identification of patients with rapidly progressive disease, and to grant access to chemotherapy in the event that an adjuvant one could not be administered in the post-surgical phase [11]. Considering the great variability of the studies in terms of CRT protocol and patient selection, is not clear in literature what proportion of patients become effectively resectable after downstaging therapy [12]. Actually, radiological assessment of residual PDAC after neoadjuvant CRT represents one of the major radiological challenges in pancreatic cancer management [12]. It has long been known that computed tomography (CT), despite being the method of choice for the staging of PDAC, tends to be less accurate in the evaluation of the effective post-CRT resectability, reporting an overestimation of real tumor extension in most of the studies [13,14,15]. The mismatch between radiological and histological response in the downstaging assessment is mainly due to the anatomopathological nature of PDAC, which consists of a great number of tumor cells embedded in a dense fibrotic stroma. After CRT, tumor cell numbers may be reduced, but the preexisting fibrous matrix persists, with the eventual addition of further fibrosis and a variable quote of perilesional edema, mimicking pathologic tumor tissue persistence [16]. Even inflammatory remodeling following bile duct drainage can compromise the downstaging assessment [12]. With this paper, we attempt to identify the most reliable imaging parameters in our multi-center population for the correct evaluation of the PDAC downstaged after neoadjuvant CRT, in particular regarding the most common qualitative and quantitative imaging criteria in comparison with the anatomopathological specimen. 

## 2. Methods

### 2.1. Patients Population

We retrospectively enrolled patients between February 2013 and July 2017 with a diagnosis of borderline resectable or locally advanced PDAC in agreement with Society of Abdominal Radiology and American Pancreatic Association criteria [17] after the end of their downstaging therapy with neoadjuvant CRT. Patients were selected from the revision of radiological, oncological, surgical, and anatomopathological databases of the Pancreas Institute from Policlinico “G.B. Rossi” of Verona, “Sacro Cuore—Don Calabria” Hospital of Negrar, and “P. Pederzoli” Hospital of Peschiera del Garda. The inclusion criteria were: patient underwent radical surgery after response to neoadjuvant CRT; availability of the anatomopathological specimen; availability of a contrast-enhanced CT or magnetic resonance (MR) performed between the end of neoadjuvant CRT and within 3 months before surgery. 

### 2.2. Imaging Technique

CT was performed by using two multi-slice equipment 64-detector rows (Brilliance 64; Philips Healthcare, Best, The Netherlands. Perspective 64; Siemens Healthcare, Erlangen, Germany). Scans were acquired before and after 1.5 mL/kg intravenous injection of iodine contrast media (Ultravist 370, Schering, Berlin, Germany) during pancreatic arterial phase (15s after aortic bolus-tracking peak), venous phase (60–70s), and equilibrium phase (5 min). Section thickness was 2 mm, kV 120, and mAs 125–250. MR was performed by using a 1.5T equipment (Avanto, Siemens Healthcare, Erlangen, Germany), including the following pulse sequences: axial fat-suppressed T1-weighted gradient-echo; axial/coronal T2-weighted half-Fourier acquisition single-shot turbo spin-echo (HASTE); axial fat-suppressed T2-weighted turbo spin-echo; single shot HASTE MR cholangiopancreatography. Diffusion-weighted images were acquired using echo-planar imaging sequences with three b-values (50, 400, 800 s/mm^2^). Dynamic imaging was performed with a volumetric 3D gradient-echo pulse sequence (volume interpolated breath-hold examination) with fat saturation on the axial plane, during injection of 0.2 mL/kg of Gd-BOPTA (MultiHance, Bracco, Milan, Italy) with the same phases described for CT evaluation. 

### 2.3. Imaging and Anatomopathological Evaluation

Imaging parameters have been evaluated by three radiologists: one radiologist with more than 18 years’ experience in pancreatic imaging, one middle-expert general radiologist from a different institute, and one young radiologist who trained in our pancreatic high-volume center. Quantitative parameters evaluated were: tumor dimensions in the three common orthogonal axes; tumor major axis; tumor volume. Main qualitative parameters evaluated were: tumor location; tumor enhancement compared to pancreatic parenchyma in arterial, venous, and equilibrium phases; enhancement homogeneity; tumor persistence (hypovascular non-fibrous tissue inside the lesion); fibrosis presence; main pancreatic duct dilatation >5 mm; retroperitoneal infiltration; involvement of portal vein, celiac trunk, mesenteric artery, mesenteric vein; perivascular cuff (hypodense avascular undefined tissue around celiac trunk and major vessels). Tumor dimensions, persistence, fibrosis, retroperitoneal infiltration, and presence of surgical margin infiltration (R+) have been subsequently evaluated on the operative sample by an expert pathologist with decades of experience in pancreatic tumors.

### 2.4. Statistical Analysis

Imaging and anatomopathological parameters were categorized into dichotomous classes. Different dimensional cut-offs were considered with an increase of 5 mm to categorize the tumors according to their major axis. Single radiological evaluations and consensus evaluation have been tested. Consensus evaluation was considered as the most frequent measure among the three single evaluations of the same parameter for qualitative measures and as the mean value for quantitative measures. The single evaluation from pancreas-master radiologist was taken as the imaging gold standard and compared to consensus evaluation. Accuracy, sensitivity, and specificity compared to anatomopathological results were evaluated for each parameter. Chi-Square test was used to assess correlations between categorical variables. A logistic regression model was used to calculate the risk of R+ resection related to the dimensions at imaging. Cohen’s K-coefficient has been calculated between single evaluations to assess the inter-observer agreement (IOA). Continue variables with normal distribution were expressed as mean ± standard deviation, while median (interquartile range) was used for non-normal distributions. Statistical analysis was performed by using Analyse-it software for Microsoft Excel (Analyse-it Software, version 4.51, Ltd, http://www.analyse-it.com/; 2009) and IBM SPSS Statistic (v.19).

## 3. Results

### 3.1. Population and Tumour Characteristics

In total, 71 patients met the inclusion criteria and have been included in the study. All major population characteristics are summarized in Table 1. The mean age of patients was 63.8 ± 8.4 years and 45.1% were males. Further, 78.9% of patients have been studied with CT, 21.1% with MR, while 45.1% of patients received FOLFIRINOX CRT, 32.4% Gemcitabine-Abraxane, 2.8% FOLFIRINOX-radiotherapy, 2.8% Gemcitabine-Abraxane-radiotherapy, and 16.9% received a combination of previous mentioned chemotherapeutic agents. Additionally, 78.9% of patients underwent duodenocephalopancreatectomy, 15.5% splenopancreatectomy, and 5.6% total pancreatectomy. 

All major findings from imaging and anatomopathological evaluation are summarized in Table 2. At the consensus evaluation, 97.7% of lesions were >15 mm cut-off, 81.8% were >20 mm, 40.9% were >25 mm, and 20.5 were >30 mm. Median major axis dimension was 22.3 (18.7–27) mm at consensus evaluation compared to 23.5 (15–30) mm at anatomopathological observation. The majority of lesions were located in the head-isthmus of the pancreas and in the uncinate process, both at imaging and histology. The strong minority of the lesions showed positive enhancement compared to the healthy pancreatic parenchyma, both at the single and consensus assessment. Significant discrepancies were reported in the evaluation of the homogeneity pattern in the three contrast-enhanced phases between the single and the consensus evaluation. Most lesions showed fibrosis both at imaging and at pathology observation. The perivascular cuff was present in about one-third of imaging evaluations. Tumor persistence was shown in half of the cases at the imaging evaluation and then turned out to be much more frequent in the surgical specimen. Surgical resection margins were mostly infiltrated at the post-operative evaluation, with 62% of R1 and only one patient with R2 resection, although more than half of the cases did not demonstrate retroperitoneal involvement.

### 3.2. Imaging and Anatomopathological Correlation

There were no significant differences in sensitivity, specificity, and accuracy between the single pancreatic-expert radiologist and consensus lecture, although there was always a slight tendency for consensus reading to be more accurate. Sensitivity and accuracy in evaluating real tumor dimensions progressively decreased with the increment of the dimensional cut-off. 

**Figure 1:**
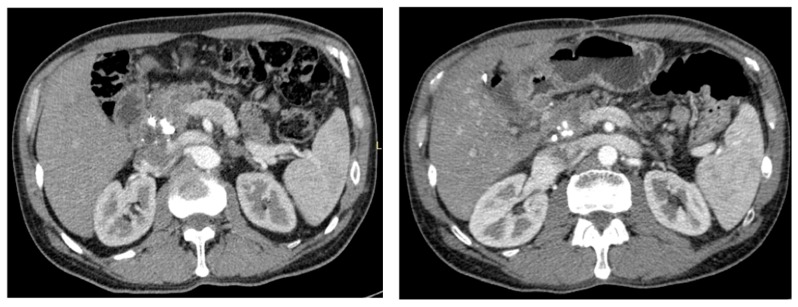
CT images of PDAC (pancreatic phase). At the CT evaluation; the lesion before chemoradiotherapy (left) is quite similar to the post-chemoradiotherapy evaluation (right), showing only minimal dimensional regression. No tumor persistence was reported in the resection specimen.

The reported accuracy values for the imaging consensus evaluation of fibrosis were 64%, 62% for tumor persistence (Figure 1), and 64% for retroperitoneal involvement. All sensitivity, specificity, and accuracy values reported for imaging parameters referring to the respective anatomopathological findings are summarized in Table 3.

The surgical resection margin R classification was statistically correlated to the size of the lesion when categorized above or below the 25 mm cut-off at imaging, both when measured by the pancreatic-expert radiologist (*p* = 0.001) and at consensus evaluation (*p* = 0.037). In total, 78% of lesions with R0 resections were <25 mm at pancreatic-expert radiologist evaluation, while 65% of R1 resections were >25 mm. Only 14% of tumors >25mm at imaging led to an R0 resection.

The 25 mm cut-off was 67% sensitive, 90% specific, and 77% accurate in assessing real tumor dimensions. The 25 mm cut-off was the only one capable of reaching values of accuracy >50% in assessing tumor persistence (53%), retroperitoneal infiltration (58%), and surgical margin infiltration (69%). Referring to R+ surgical margin infiltration, the 25 mm cut-off was 64% sensitive and 78% specific. Moreover, sensitivity and specificity for 25 mm cut-off were, respectively, 23% and 94% related to retroperitoneal infiltration, and 51% and 67% related to tumor persistence (Table 4). 

When measured by a pancreatic expert radiologist, each 5 mm increase in tumor dimension reported an OR of 1.79 (95% CI 1.13–2.80, *p* = 0.012) for R+ resection, with an of OR 6.56 (95% CI 2.07–20.81, *p* = 0.001) when bigger than 25 mm. Even at consensus evaluation, lesions >25 mm reported an OR of 3.39 (95% CI 1–11.58, *p* = 0.05) for a R+ resection.

**Figure 2:**
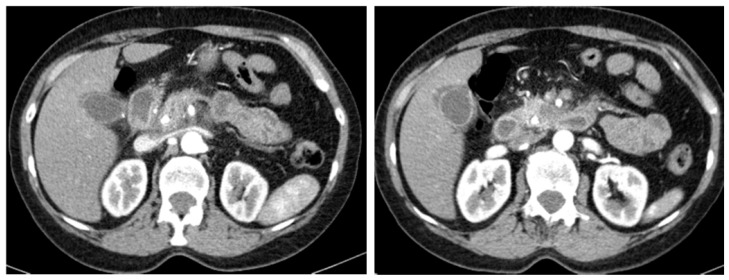
CT examination (pancreatic phase) of ductal adenocarcinoma of the pancreatic head with the presence of the perivascular cuff surrounding the superior mesenteric artery.

The presence of the perivascular cuff at imaging (Figure 2) evaluation reported a 30% accuracy in determining tumor persistence and retroperitoneal margin infiltration in the anatomopathological specimen. No statistical correlation was reported between the presence of the perivascular cuff and R+ resection, both at pancreatic-expert evaluation (*p* = 0.362) and consensus (*p* = 0.098). Whereas tumor density homogeneity in venous and equilibrium phase reported an accuracy of 50% and 63% respectively in evaluating tumor persistence, tumor enhancement in both phases was not an accurate parameter (19% and 28%, respectively). However, the presence of enhancement in the venous and equilibrium phase was found to be an accurate measure of the presence of fibrosis, with respective accuracy of 95% and 89% in consensus lecture.

### 3.3. Interobserver Agreement

Concordance values between the three radiologists are summarized in Table 5. For dimensional assessment, the concordance between individual evaluations has progressively increased from poor to moderate with the increment of the dimensional cut-off. IOA was always poor in assessing the enhancement of lesions and its homogeneity after the administration of contrast media. The evaluation of fibrosis presence and retroperitoneal involvement was poor to fair too. The concordance in assessing the tumor persistence was fair to moderate. Most of the time IOA was higher between the pancreas expert and young radiologist than between the pancreas expert and general radiologist coming from a different institute. Good IOA was reported between the pancreatic expert radiologist and consensus evaluation regarding 25 mm cut-off (k = 0.61) and perivascular cuff presence (k = 0.69).

## 4. Discussion

Imaging is of fundamental importance in the identification, characterization, and staging of PDAC. One of the biggest challenges in pancreatic imaging is to correctly restage a locally advanced PDAC after downstaging CRT to assess its resectability. In 2001, White et al. [14] first noticed how CT tends to overestimate the extension of a locally advanced PDAC subjected to neoadjuvant-CRT. The overestimation is due to multiple factors, in particular to the presence of peripancreatic edema and fibrosis induced by CRT, which can mimic a residual tumor. In 2009, Kim et al. [18] confirmed how neoadjuvant CRT decreases the accuracy of PDAC restaging, however, not enough to affect its resectability. Soon after, Morgan et al. [19] showed how patients who underwent neoadjuvant-CRT and were judged to be resectable at imaging reported higher overall survival than unresectable patients and resectable patients not treated with CRT. Therefore, they stressed that it was essential to achieve high values of accuracy on the determination of resectability post-CRT, suggesting not to overestimate the vascular involvement in order not to deny surgery to potentially resectable patients. In their study it also emerged that in patients undergoing CRT, the IOA drastically dropped in staging and in the determination of resectability compared to patients not treated with CRT. They suggested that probably the reader’s knowledge of whether the patient underwent CRT or not which could be a bias for resectability determination, a finding supported by the fact that the resectable patients were more in the CRT group than in the control. In our study, every patient underwent neoadjuvant-CRT according to inclusion criteria, so we can confirm that IOA in evaluating tumor characteristics is generally poor to fair in those patients. Pre-CRT CT or MR was not considered in our study. If, on the one hand, the comparison with the previous CT can be helpful, there is the risk of provoking a misleading enthusiasm in the reader, affecting its determination of resectability. For example, a dimensional reduction of a lesion does not essentially mean its resectability and needs to be considered separately. In fact, as shown by Cassinotto et al. [20], changes in tumor major diameter are not always associated with R0 resection. Moreover, Katz et al. [21] demonstrated that current radiological measures of CRT response, such as Response Evaluation Criteria In Solid Tumor (RECIST) [22], are not reliable in evaluating effective resectability of PDAC. To blind the radiologist on the neoadjuvant-CRT carried out or on the previous CT or MR study in order to avoid misleading evaluations in our opinion is not appropriate in clinical practice, but should be useful to research what are the most common factors not to be overestimated at preoperative imaging to ensure greater accuracy and a good IOA on the judgment of resectability. In the setting of this study, we wanted to consider only patients subjected to CRT in order to better understand which parameters are the most reliable in the evaluation of this type of patient. Moreover, pre-downstaging imaging, and therefore tumor shrinkage, was not considered in order not to influence readers on the actual tumor size. 

Probably one of the most misleading causes of underestimation of PDAC resectability is the persistence of perivascular hypodense tissue after neoadjuvant-CRT. This perivascular cuff could better rely on lymphatic congestion or CRT-related fibrotic rehash than on tumor infiltration. Indeed, in our study the presence of the perivascular cuff was not accurate in assessing tumor persistence and was not statistically correlated with surgical margin infiltration and R+ resection (*p* = 0.362), therefore we suggest giving it little consideration in resectability evaluation. In the same way, as proven by Cassinotto et al. [13], some degrees of portal and superior mesenteric vein strain and stenosis could persist after CRT due to fibrotic tissue, but do not necessarily mean that tumor is still infiltrating the vessels. 

Despite the homogeneity of tumor density in the venous and equilibrium phase being quite accurate in evaluating tumor persistence, it was not useful in assessing its resectability in our study. Our finding confirms that one should not rely on tumor density and enhancement to predict R0 resections, because imaging cannot effectively differentiate tumor from necrotic or fibroinflammatory tissue post-neoadjuvant CRT [20]. 

More than half of our patients presented margin infiltration at histological evaluation of surgical specimen, probably due to a high component of borderline resectable PDAC in our population. However, the near totality of the R+ was R1, and therefore reported a solely microscopic surgical margin infiltration. For this reason, we could always consider the size and the extent at the anatomopathological evaluation coinciding with the real measurements of the tumors. In our study, the strong majority of R0 tumors were smaller than 25 mm, while about half of R+ tumors were >25 mm, with a significant statistical difference both at pancreatic expert radiologist (*p* = 0.001) and consensus evaluation (*p* = 0.037). Testing different major axis cut-offs, we found that there is no one capable of simultaneously maximizing sensitivity, specificity, and accuracy referring to anatomopathological tumor diameter, persistence, and retroperitoneal margin infiltration. Nevertheless, we found how 25 mm imaging cut-off is accurate in assessing real tumor dimension, reporting moderate IOA. Referring to retroperitoneal infiltration, 25 mm imaging cut-off is 94% specific, 58% accurate, but only 23% sensitive. However, when considering overall surgical margin infiltration, the 25 mm cut-off was 64% sensitive, 78% specific, and 69% accurate in predicting an R+ resection. These mean that about 70% of the times, the 25 mm cut-off can correctly diagnose the R0 or R+ of the surgical resection. In particular, if tumor dimensions are evaluated by an expert radiologist, each increase of 5 mm leads to a 79% increase in R+ risk (*p* = 0.001), reporting a higher risk for tumors >25 mm with an OR of 6.56 (95% CI 2.07–20.81, *p* = 0.001). 

All these findings support the argument to be more aggressive in borderline resectable PDAC multimodality treatment, as previously suggested in literature, in order to not deny surgery to a potentially resectable patient [19,20,21]. In our multi-center experience, common criteria of tumor resectability cannot be fully applicable to PDAC after neoadjuvant chemotherapy, due to overestimation of tumor extension by imaging studies. We suggest using a multiparametric approach to resectability evaluation after CRT. In particular, surgery must be considered for tumors which after CRT are smaller than 25 mm, with reduction of vascular contact, independently from the persistence of the perivascular cuff or inhomogeneous enhancement. 

A final interesting fact is that IOA mostly tended to be higher between the pancreas expert and young radiologist who did all their residency period in a pancreatic institute, than between the pancreas expert and general older radiologist coming from a different non-specialized institute. Most of the time the pancreatic expert radiologist agreed with young radiologist but disagreed with the older general radiologist. For this reason, consensus evaluation often coincided with the expert’s evaluation and preserves diagnostic and statistical significance. This underlines how radiological specific expertise is essential in a highly specific ambit, such as pancreatic diseases, and more important than years of working experience and is independent from overall radiological knowledge.

The retrospective setting was the main limitation of the study. The moderately low number of patients who were included in the analyzes may represent an additional limitation. Further prospective studies on larger series are needed in the future to confirm the findings in this study. Furthermore, comparison studies between downstaging and upfront resection will be useful in the future, eventually considering preoperative imaging and tumor shrinkage.

## 5. Conclusions

Common radiological criteria are not fully applicable to assess resectability of PDAC after CRT. Basically, imaging methods tend to underestimate PDAC resectability after neoadjuvant-CRT. IOA is poor to fair in evaluating most of the qualitative parameters of downstaged pancreatic adenocarcinoma. Surgery should be considered for downstaged borderline resectable PDACs, independently from perivascular cuff presence, especially for tumors smaller than 25 mm.

## Figures and Tables

**Table 1:** Main characteristics of the study population.

Study Population (N = 71)	Values
Age (years)	63.8 ± 8.4
Male (%)	45.1
CT evaluation (%)	78.9
MR evaluation (%)	21.1
FOLFIRINOX (%)	45.1
Gemcitabine-Abraxane (%)	32.4
Other CRT protocol (%)	22.5
Dudoenocephalopancreatectomy (%)	78.9

**Table 2:** Characteristics of lesions at PER single evaluation and consensus evaluation, compared to the anatomopathological findings. PER = Pancreas Expert Radiologist. AP = Anatomopathology.

Lesion Parameters (N = 71)		PER	Consensus	AP
Major Axis (mm)		25 (20–30)	22.3 (18.7–27)	23.5 (15–30)
Homogeneous Pattern (%)	Arterial	28.5	65.1	−
	Venous	88.3	50	−
	Equilibrium	26.5	58.8	−
Enhancement (%)	Venous	4.8	1.59	−
	Equilibrium	21.9	9.4	−
Perivascular Cuff (%)		36.6	26.8	−
Tumor Persistence (%)		49.3	47.9	78.9
Retroperitoneal Infiltration (%)		43.7	29.6	23.9
Fibrosis (%)		64.1	71.8	57.8
R+ Resection (%)		−	−	63.4

**Table 3:** Sensitivity, specificity, and accuracy values reported for imaging parameters referring to the respective anatomopathological finding. AC = Accuracy. SE = Sensitivity. SP = Specificity.

Lesion Parameters (N = 71)		SE (%)	SP (%)	AC (%)
Major Axis	>20 mm	93	46	79
	>25 mm	67	90	77
	>30 mm	31	86	66
Retroperitoneal infiltration		53	68	64
Fibrosis		50	65	64
Tumor Persistence		64	57	62

**Table 4:** Sensitivity, specificity, and accuracy values reported for 25 mm cut-off in reference to the anatomopathological margin infiltration and tumor persistence. AC = Accuracy. SE = Sensitivity. SP = Specificity.

25 mm vs.	SE (%)	SP (%)	AC (%)
Surgical margin infiltration (R+)	64	78	69
Retroperitoneal infiltration	23	94	58
Tumor Persistence	51	67	53

**Table 5:** IOA (Cohens’ k coefficient) among the three radiologists in the evaluation of single imaging parameters. IOA = Interobserver Agreement. PER = Pancreatic Expert Radiologist. GR = Middle-expert General Radiologist. YR = Young Radiologist.

Parameters		PER vs. GR (k)	PER vs. YR (k)	GR vs. YR (k)
Major Axis	>15 mm	−0.03	0.31	−0.07
	>20 mm	0.18	0.39	0.32
	>25 mm	0.33	0.55	0.46
	>30 mm	0.48	0.53	0.54
Homogeneous pattern	*Arterial*	−0.03	0.17	0.24
	*Venous*	0.08	0.08	0.02
	*Equilibrium*	0.09	0.24	0.18
Enhancement	*Venous*	−0.03	−0.04	−0.04
	*Equilibrium*	−0.09	−0.29	0.21
Retroperitoneal infiltration		−0.03	0.28	0.39
Fibrosis		0.28	−0.11	0.27
Perivascular Cuff		0.29	0.59	0.46
Tumor Persistence		0.28	0.48	0.38

## Abbreviations

CT	Computed Tomography
CRT	Chemoradiotherapy
HASTE	Half-Fourier Acquisition Single-shot Turbo spin-Echo
IOA	Inter-Observer Agreement
MR	Magnetic Resonance
OR	Odds Ratio
PDAC	Pancreatic Ductal Adenocarcinoma
R0	No Surgical Margin Infiltration
R+	Positive Surgical Margin Infiltration
